# The Role of A Priori Belief in the Design and Analysis of Fault-Tolerant Distributed Systems

**DOI:** 10.1007/s11023-023-09631-3

**Published:** 2023-04-17

**Authors:** Giorgio Cignarale, Ulrich Schmid, Tuomas Tahko, Roman Kuznets

**Affiliations:** 1 TU Wien, Embedded Computing Systems Group, Vienna, Austria; 2 Department of Philosophy, University of Bristol, Bristol, UK

**Keywords:** Distributed Systems, Philosophy of Computation, A Priori Knowledge, Fault-tolerant Systems

## Abstract

The debate around the notions of a priori knowledge and a posteriori knowledge has proven crucial for the development of many fields in philosophy, such as metaphysics, epistemology, metametaphysics etc. We advocate that the recent debate on the two notions is also fruitful for man-made distributed computing systems and for the epistemic analysis thereof. Following a recently proposed modal and fallibilistic account of a priori knowledge, we elaborate the corresponding concept of a priori belief: We propose a rich taxonomy of types of a priori beliefs and their role for the different agents that participate in the system engineering process, which match the existing view exceedingly well and are particularly promising for explaining and dealing with unexpected behaviors in fault-tolerant distributed systems. Developing such a philosophical foundation will provide a sound basis for eventually implementing our ideas in a suitable epistemic reasoning and analysis framework and, hence, constitutes a mandatory first step for developing methods and tools to cope with the various challenges that emerge in such systems.

## Introduction and Overview

The notion of “a priori” is familiar to most from Kant’s famous work, but its history goes much further, at least to the Latin translations of Euclid’s *Elements*. During this long history, the distinction between a priori and a posteriori* knowledge* has seen many developments and has been the subject of an enormous amount of quite polarizing discussions. Besides knowledge, the debate also concerns related topics such as modality (Kripke, 1972; Putnam, 1978), justification (BonJour, 1997; Casullo, 2003), revisability (Burge, 1993, 1998; Kitcher, 1980), and many others; see (Casullo, 2014) for a comprehensive overview of the debate. As claimed by Casullo (2003), the relevance of the dispute goes beyond epistemology and reaches virtually any area of philosophy and the notions of “a priori” and “a posteriori” have been applied widely. In particular, a novel view of a priori knowledge was recently proposed by Tahko (2008, 2011) in the context of a broader metaphysics of science (cf. Tahko, 2015). Along the same lines, we propose that these notions can also be put to fruitful use in the field of distributed computing systems, where a priori knowledge plays a crucial role both in their design process and their epistemic analysis. *Distributed systems* (Coulouris et al., 2011; Lynch, 1996) consist of multiple computing nodes that execute their programs independently of each other. Nodes can communicate with each other, however, by sending messages over a communication network or via shared memory. In the distributed computing literature, the active entities are usually called *processes* (we will subsequently use the term *agents*) and are assumed to execute some *protocols* in the course of a *run* that constitutes a possible time-evolution of the system. Designing distributed systems is difficult due to the inherent uncertainty of any process about the actual computation speeds and message delays, for example. The design of such a system inevitably involves the need to come up with a set of *system assumptions* (like message delay bounds and failure rates) that are assumed to hold throughout the lifetime of the system. This brings us to the first point of connection between the philosophical literature and the epistemic analysis of distributed systems, since the relevant system assumptions are best understood as holding “a priori”, in the sense that they are assumed to hold irrespectively of any execution of the system. This is of course not quite the usual sense of a priori knowledge familiar from philosophical literature, i.e., knowledge that is independent of experience. But as we will see, there are striking structural similarities between this applied notion of the a priori and the philosophical analysis of the notion.

The first complication that we encounter is that distributed systems are characterized by a vast spectrum of possible behaviors, which often cannot even be exhaustively envisioned beforehand. Especially challenging is the design of *fault-tolerant distributed systems*, which must also be able to cope with (some) erroneous behavior of processes and communication. Special kinds of failures, such as byzantine failures (Lamport et al., 1982) where byzantine-faulty agents may deviate arbitrarily from their protocols, are particularly problematic, as malicious agents may start to execute actions that jeopardize the original goals of the system. Correct agents are typically not aware of the existence, goals, or capabilities of such faulty agents. To guarantee a certain degree of efficiency, such systems require even stronger *system assumptions* than fault-free systems. Such system assumptions need to be provided and made available to the processes by the system designer during system development. Thus, through the paper, we distinguish between two different kinds of agency:

- the *system designer*, the idealized (traditionally human) designer of the system, who is responsible for its development and well-functioning and
- the processes (agents) of a distributed system that are computing devices with only a limited view of the system (compared to the view the system designer has). In particular, they can only act according to their protocols (unless faulty).

In epistemic modeling and analysis of multi-agent systems (Fagin et al., 2003), a set of processes (agents) use some protocols for solving a task, based on their limited local view of the system achieved during the execution. Their reasoning is formulated in terms of epistemic logic (Van Ditmarsch et al., 2015), where agents’ knowledge is captured via a possible world semantics (Hintikka, 1962).^1^ Such semantics is represented by relational structures called “Kripke models”, which proved to be a powerful conceptual abstraction for distributed systems: reasoning about agents’ “states of knowledge” at various points in the execution of a distributed protocol (Halpern, 2003; Halpern & Moses, 1990) turned out to be extremely fruitful both for the designer and the analyzer of a distributed system. As usual in epistemic logic, agents are logically omniscient, meaning that they are able to derive all logical consequences of the formulas they already know to be true. Nonetheless, their limited view of the system leads to considerable uncertainty regarding which of all the possible states represents the actual state of affairs. Furthermore, their knowledge is inherently limited to the set of states that are considered possible in the system, which are themselves bound by the system assumptions formulated by the system designer.

We argue that this set of system assumptions may be interpreted as constituting the core of the system’s a priori knowledge and that the underlying notion is *fallibilistic and modal.* This is, of course, a clear departure from the traditional notion of a priori knowledge, but in the philosophical landscape, such a view was already proposed by Tahko (2008, 2011), along with a bootstrapping relation that captures the interplay of a priori knowledge with its a posteriori counterpart. While this usage of the notion of “a priori” is non-standard in contemporary epistemology, we propose that the applied and much more restricted context of man-made distributed systems provides a less controversial arena for demonstrating the fruitfulness of Tahko’s original ideas, which we discuss in detail in the next section. In this sense, the proposal can be seen as engaging in a form of “conceptual engineering”, i.e., fixing or adapting an old concept for a new application (Chalmers, 2020). We apply Tahko’s view to the present case and suggest that the a priori knowledge of both the system designer and especially of processes is modal and fallible; hence, we use the term a priori* belief* to reflect this:

- We use the notion of the a priori in the modal sense introduced by Tahko, i.e., related to possibility. In distributed settings terminology, we use the term “a priori” to refer to the set of states of the system that are considered possible by the agent in question.
- We use the term “belief”, combining Tahko’s interpretation of fallibility and the usage of the term in epistemic logic, i.e., we will use belief over knowledge, since there is no guarantee that such “knowledge” faithfully describes the actual world, i.e., that it is factive^2^ in the usual sense of epistemic logic terminology.
- We will call the knowledge (resp. belief) that is obtained by the agents during protocol execution “a posteriori knowledge”^3^ (resp. a posteriori belief). Thus, we use the term “a posteriori knowledge” interchangeably with “epistemic knowledge”, which is used in the field of epistemic logic. The corresponding notion for the system designer will be introduced in Sect. 3.

*Main contributions.* By adapting the ideas formulated by Tahko (2008, 2011) to distributed settings, we bring to light the various forms and usages of a priori beliefs hidden in the design process of distributed systems, while explaining the differences between the a posteriori knowledge obtained by the processes during their protocol execution and the a priori beliefs built into these protocols. We also identify the major differences of our applied account of a priori knowledge from the account by Tahko (2008, 2011).

Moreover, we substantiate the conjecture, which has its roots in Kuznets et al., (2019a, 2019b), that explicitly incorporating a priori beliefs in the epistemic analysis of *fault-tolerant* distributed systems is of utmost importance: the presence of faults may provide agents false information that contradicts their a priori beliefs (e.g., by violating system assumptions such as bounds on message delays), thus, forcing a correct agent to mistakenly believe itself (or some other agent) to be faulty. Given the elevated number of possible (faulty) behaviors, an epistemic Kripke model encompassing all of those would be simply too vast to be of any use. By contrast, the system designer can detect such mistakes during runtime and can subsequently force another iteration of the design cycle, where she updates agents’ a priori beliefs (corresponding to the set of states considered possible in the Kripke model), by extending the set of reachable states when needed. This way, there is no need to foresee and cover all possible (faulty) behaviors beforehand.

To facilitate this goal, we provide a rich taxonomy of a priori beliefs, and distinguish their role and use with respect to the agents and the kind of systems considered. Ultimately, a clear separation between what is computed based on the actual state of affairs (a posteriori knowledge) and what is considered locally possible (a priori beliefs) by agents not only sheds light on the pivotal role of a priori beliefs in the design process, but also crucially adds explanatory power to the epistemic analysis of distributed systems.

We emphasize, however, that the focus of our paper is on the *philosophical foundations* and not on possible approaches for *implementing the resulting ideas* in some epistemic reasoning framework. In more detail, whereas we use epistemic terms and notations for exhibiting the actual linkage points with the philosophical concepts and in some of our examples, it is outside the scope of our paper to consider possible ways of implementing, e.g., a priori reasoning and model updates. We will leave this important next step to our future work.

*Paper organization.* In Sect. 2, we consider Tahko’s account of a priori knowledge and its relationship with a posteriori knowledge, constituting the philosophical background on which our proposal is based. In Sect. 3, we sketch the design process of a distributed system, providing the background knowledge necessary to understand where a priori beliefs enter the picture; we elaborate the use of a priori beliefs in distributed systems during the *Development Phase*. In Sect. 4, we discuss the role of a priori beliefs during the *Operating Phase* after the system is deployed, and we explicitly relate our inquiry on a priori beliefs to epistemic modal logic (Sect. 4.1). At the end of each section (Sects. 3.1 and 4.2), we discuss the similarities and differences w.r.t. the account presented by Tahko (2008, 2011). Finally, in Sect. 5 we conclude by summarizing the contributions of this paper in (i) explicating the role of a priori beliefs in the design of distributed systems and (ii) highlighting its relevance in their epistemic analysis.

## The Modal and Fallibilistic Account of A Priori Knowledge

In the standard view, the concepts of a priori and a posteriori are mutually defined by their opposite relation with respect to the notion of experience: the former is defined as independent of experience, while the latter relies on experience (Kitcher, 1980 p. 4). However, the distinction between the two notions is not as sharp as it might seem at first, and influential philosophers (Quine, 1951; Williamson, 2013) have problematized it. For instance, Williamson observes a problem with the fact that, traditionally, every specific way of knowing has been taken to be either a priori or a posteriori, and not both. This introduces a problem because there are cases where we seem to be able to come to know the same proposition both by a priori and a posteriori means. Accordingly, we would need to refine the distinction if we don’t want the same piece of knowledge to be both a priori and a posteriori. Williamson speculates that ‘the best fit to current practice with the term is to stipulate that a truth is a posteriori if and only if it can be known a posteriori but cannot be known a priori’ (2013 p. 293). But as Williamson goes on to point out, Kripke’s (1972) famous examples of contingent a priori truths and necessary a posteriori truths may cause further problems here. In particular, if there are contingent a priori truths, such as Kripke’s example concerning the standard meter, then it seems that they must also be knowable a posteriori. This severs the link between apriority and necessity. The upshot for Williamson is that the differences between a priori and a posteriori knowledge are superficial because experience plays a role for both kinds of knowledge, and he argues that this role is not just ‘purely enabling’—like it would perhaps be in the case of concept acquisition—while also being less than ‘strictly evidential’, such as in the case of pure perceptual information.

We accept Williamson’s challenge as it applies to the traditional distinction between a priori and a posteriori knowledge and acknowledge that this requires a revision of these concepts. However, the discussion has grown enormously and proponents of the distinction have related it to other notions rather than to experience. Among these, Tahko (2008, 2011) claims that the key concept grounding the a priori is “modality”; more precisely, metaphysical modality (i.e., possibility and necessity). In light of Kripke’s argument against the link between apriority and necessity, this move may seem ill-advised, as we have just seen with reference to Williamson. Instead, Tahko posits that Kripke’s analysis does not rule out the connection between apriority and metaphysical modality in general, but only between apriority and necessity. In his view, the a priori and metaphysical modality can still relate via the notion of possibility. So, on this view, a priori reasoning is thought to concern metaphysical possibilities. In Tahko’s view, this enables an analysis of a priori and a posteriori knowledge that retains some aspects of the traditional conception while giving up the sharp distinction in terms of experience that Williamson focuses on. However, for our current purposes, an even more contained version of the distinction can be applied, as the domain of a priori knowledge is solely set by the system designer and determines, e.g., via system assumptions, what agents could possibly experience in a run. As we will argue throughout the paper, while agents’ a priori knowledge determines what they can possibly experience, only what the agents actually experience in a run may constitute agents’ a posteriori knowledge which is thus clearly separated from the former.

Viewed more abstractly, a priori reasoning operates in the realm of metaphysical possibility and determines the range of different possible scenarios that are consistent with the data currently at hand. In Tahko’s account, a priori reasoning is not supposed to be mere conceivability, nor is it based on intuitions or any special rational capacity. Rather, a priori reasoning is considered a fairly mundane capacity that rational agents have, closely analogous to the capacity to formulate a scientific hypothesis. Accordingly, this is especially fitting for the process of scientific discovery, to which Tahko explicitly refers (2011, 2020), where the aim is to determine what is true in the actual world. In this process, the role of the a priori is to determine the range of possibilities that need to be tested by empirical means. We do not need to take a stand here regarding the nature of a priori reasoning, as it is sufficient for the present application to consider the much more restricted case of processes (agents) of a distributed system, and such agents gain their initial a priori “knowledge” more directly from the system designer. The definition of apriority that emerges from Tahko’s account is something like the following (after Tahko, 2008 p. 60):

**A Priori Knowledge.** *Any metaphysically possible, logically consistent a priori proposition may constitute ‘a priori knowledge’, whether or not it is true in the actual world.*

The modal element of Tahko’s view should be clear from this, but what about the fallibility? In fact, Tahko suggests that there are two senses of fallibility at play here; one is human error, in which case the proposition wasn’t really a priori to begin with (say, because it was not consistent). Whereas this is, of course, a source of potential error, we consider it as something that we can safely ignore in our considerations as (i) it is way less likely to occur compared, e.g., to the wealth of possible programmer’s errors and (ii) in the present context, nothing could be done about it anyway. On the other hand, insofar as the proposition is a priori, it would always correspond with a metaphysical possibility. So, in the latter case, the fallibility just concerns the question of whether the metaphysical possibility captured by the proposition is also actual (or factive). It’s this aspect of Tahko’s account that turns out to be the most useful for the present application, as we will detail below: the agents in a system may be considered to be fallible in a structurally similar sense. What this means in the context of Tahko’s account is that we never falsify the a priori metaphysical possibility, but we may falsify its actuality (say, because new empirical evidence rules out something we thought might be actual but was merely possible). In this paper we will focus on this latter sense of fallibility, as it is concerned with a priori fallible propositions and not with a posteriori propositions mistakenly considered a priori because of human error.

A key upshot of the framework is that there is a constant interplay between the a priori and the a posteriori, in what Tahko calls a *bootstrapping relationship*, “A priori knowledge generally advances in very small steps: we introduce an a priori proposition, which we then attempt to verify by a posteriori means; this is the core of the bootstrapping relationship” (2011 p. 152). Tahko’s main example of this relation concerns the phenomenon of gravitational redshift^4^ in the history of science (2011 p. 152). Gravity’s effect on light was a phenomenon envisioned by Newton’s theory, but the results were inaccurate due to Newton’s assumptions about the corpuscular nature of light. By assuming the wave nature of light and the possibility of change in time flow, Einstein’s general theory of relativity, on the other hand, was able to obtain more accurate predictions. In a nutshell, Tahko claims that the dynamics behind this process involve a priori reasoning and a posteriori verification.

**Fig. 1:**
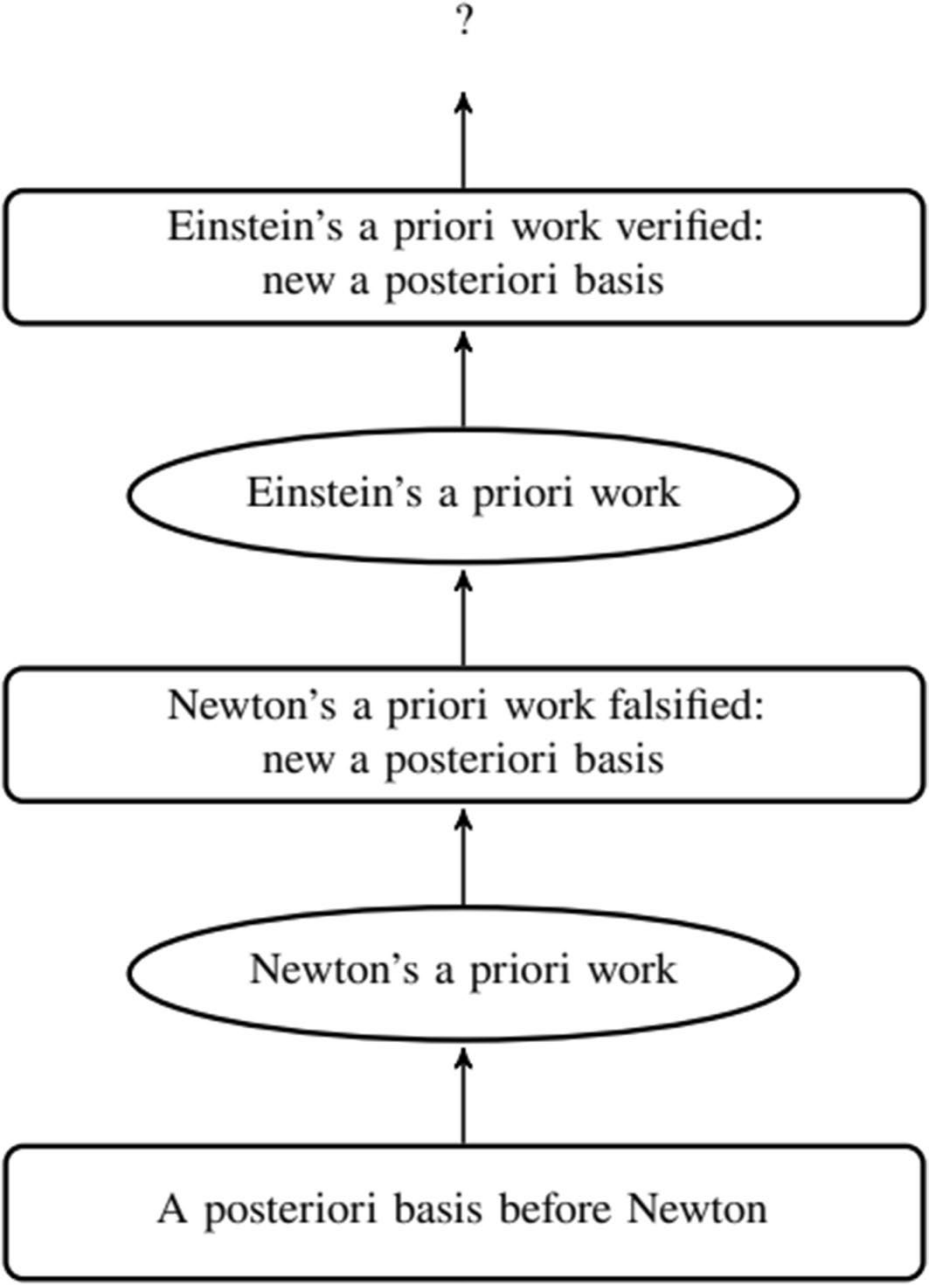
The bootstrapping relation

The process is represented in Fig. 1. The first step, at the bottom, is the a posteriori basis representing all the knowledge about the gravitational redshift before Newton, together with the respective assumptions on the nature of light. Newton’s theory of gravitational redshift represents an a priori step in the bootstrapping sequence that introduces a new hypothesis to the framework. Effectively, an a priori step is the introduction of a proposition that is believed to be metaphysically possible. This hypothesis is then tested against the a posteriori basis: as a result, it is established that although the hypothesis does provide a better explanation of physical phenomena, it fails to coherently explain all of them. This way, Newton’s theory is incorporated into a new a posteriori basis, becoming part of the adopted a priori beliefs about the actual world. This is the process by which Tahko suggests knowledge to accumulate, i.e., a verified a priori proposition becomes part of the established a posteriori basis. Einstein’s work constitutes the next a priori step, starting out from the established post-Newton a posteriori basis, and, once Einstein’s theory is empirically verified, it is incorporated into a new (and more sophisticated) a posteriori framework.

This presentation is, of course, a simplification of the real process, and it does not include all the single instances of the bootstrapping relation (i.e., the many hypotheses and experiments conducted by different people). Nonetheless, it shows the interplay between the a priori propositions intended to explain a certain phenomenon and the a posteriori framework that effectively puts these a priori hypotheses to a test. As pointed out by Tahko (2008 p. 66), the process of a priori reasoning is not simple guessing because the new a priori propositions are based on the a posteriori framework at hand: their aim (at least in a scientific context) is to explain phenomena that contradict the actual a posteriori basis. Note that this does not make these a priori propositions empirical or a posteriori. Rather, in a scientific context we generally (although not always) delimit our attention to those a priori propositions that are compatible with the existing a posteriori basis. If the new a priori propositions prove to be (partially) correct by removing (some of) these contradictions, they can enrich the a posteriori framework, providing the basis for further development.

Given that a priori propositions are evaluated in the actual world, they are always susceptible to a falsification at a later stage: at any time, new contradictions can be discovered that invalidate (part of) the current a posteriori basis. Hence, as noted above, and following BonJour’s account (1997), Tahko claims that a priori reasoning can be fallible: a verified—or, better, not falsified—a priori proposition does not need to be metaphysically necessary: it is always conceivable that we may discover a new phenomenon falsifying it in the actual world. On the other hand, according to Tahko’s modal interpretation of a priori knowledge, the falsification of an a priori proposition cannot change its a priori status. Indeed, were a priori propositions necessary, then a falsification of one would have proven that it was not a priori in the first place. This contradiction disappears if, instead of being considered necessary, a priori propositions are viewed as restricting the set of possible scenarios. A falsified a priori proposition still retains its a priori status but has been shown *not* to hold in the actual world. At the same time, a proposition that is mistakenly considered a priori can be dismissed by showing that the proposition cannot be *possible*. Note that the verification process itself can be purely empirical: testing the actuality of a proposition is done solely by a posteriori means, without questioning the status of the proposition, but only assessing its status in the actual world.

We are not arguing here in favor of this particular view of a priori knowledge in the general philosophical context considered by Tahko. Our goals are more modest: we only claim that his notion of a priori knowledge and the bootstrapping process are well-suited for capturing the concept of a priori knowledge that is embedded in the epistemic reasoning in man-made systems.

The resulting notion of the a priori may also be fruitfully compared to Burge’s (e.g., 1993, 1998, 2010, 2020) influential work in developing the notion.^5^ Burge famously argued in favor of a moderate form of rationalism, whereby rationalism is not about ‘unrevisability, infallibility, indubitability, or innateness’ (Burge, 1998 p. 2), but rather about the force of epistemic warrant. This produces a notion of apriority that concerns justification or entitlement; an agent’s knowledge is a priori if it is supported by an a priori warrant that needs no further warrant for it to be knowledge. The view has various interesting features that are compatible with the notion of apriority adopted in this paper. In particular, Burge is adamant that a belief can have both empirical and a priori justification or entitlement and argues that a ‘justification or entitlement is apriori if its justificational force is in no way constituted or enhanced by reference to or reliance on the specifics of some range of sense experiences or perceptual beliefs’ (Burge, 1993 p. 458). This allows for considerable flexibility on what counts as a priori, and the approach is broadly compatible with Tahko’s account of the a priori. While Burge does not put forward a modal analysis of apriority, his work has helped to disentangle the notion from its traditional connotations involving unrevisability and infallibility. These are also key features of the notion of apriority that we employ. Moreover, Burge’s notion of apriority has also laid the path for a more natural use of the notion in contexts that involve non-human agents. For instance, Burge (1998 p. 4) allows that ‘knowledge that an individual obtains by being told a proposition by another person, where the individual’s warrant resides in the interlocution, can be apriori’. In the present context, the a priori knowledge of the agents comes directly from the system designer, so we obviously need a notion of apriority that is flexible in a similar fashion as Burge’s notion. Fortunately, Tahko’s approach provides a similar flexibility, with the addition of the modal element that turns out to be useful for our purposes.

## A Priori Beliefs in Designing a Distributed System

In Sect. 2, we discussed Tahko’s fallibilistic view of a priori knowledge, which is based on the notion of possibility and is in a constant interplay with a posteriori knowledge by means of the bootstrapping relation. In this section, we will provide a similar interpretation of a priori beliefs in the process of designing distributed systems and demonstrate that many aspects of his view fit very well to our specific context. On the one hand, we maintain the modal aspect of Tahko’s definition: In the present context, the modal space is determined by the set of alternative scenarios considered possible by the agent in question.

To help the reader, we will start with preliminary definitions of the terms and concepts used that will be further elaborated on later:

**System Designer A Priori Beliefs (APrBs).** *Any proposition that is meant to describe some key features of the system under consideration may constitute ‘a priori belief’, whether it actually holds or not in the current system. It comprises system assumptions, such as the basic safety and liveness specifications, and system features, such as agents’ characteristics, network topology, failure models, communication delays, encoding of messages etc. Obviously, however, there is always the possibility that these system assumptions and features may be violated in a real system, i.e., they are fallible.*

**System Designer A Posteriori Knowledge.** *Any proposition relative to the system at hand that can be inferred from an execution (or a simulation) of the system itself.*

Note that the System Designer APrBs need not be clearly and formally formulated: especially during the early phases of the design process, the system designer has only fuzzy and informal concepts in mind, and the desired result is reached by trial and error.

To exemplify the distinction, consider an ATM system in the phase of design. A basic a priori belief of the system designer in the form of a system assumption is that such a system provides money to the client if some parameters are met. Thus, the proposition “if the agent knows that the parameters are met, provide the requested amount” can be thought of as an a priori proposition that will be implemented in the system. Suppose the system is functioning. The system designer observes that some agents misbehave, i.e., the parameters are met but the money is not provided. This information constitutes a posteriori knowledge for the system designer as it is a fact inferred from the behavior of the system. This information also contradicts the system designer’s a priori belief, as it violates the proposition stated before, justifying the usage of the term “belief” instead of “knowledge”.

### System design overview

**Fig. 2:**
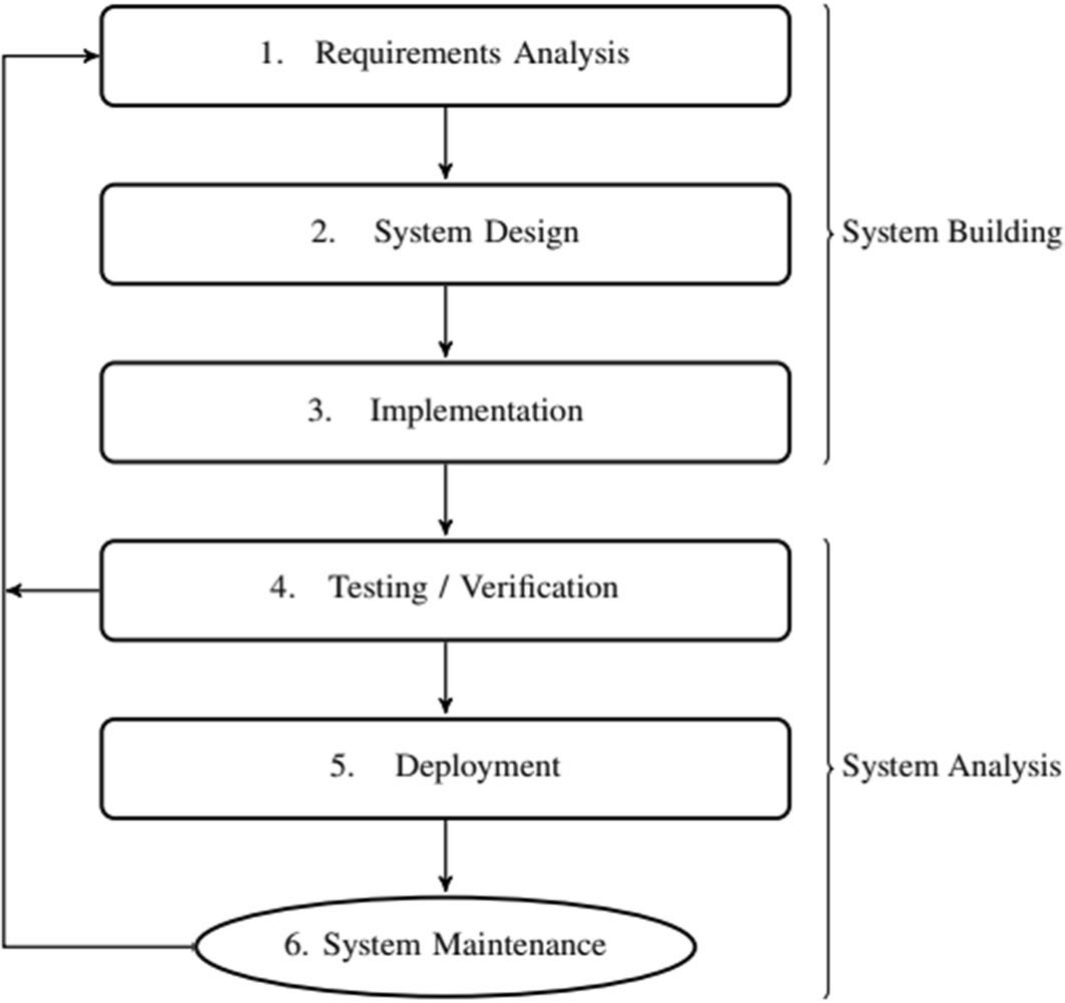
Design of a traditional distributed system

The fundamental role played by the system designer’s a priori beliefs is better captured by considering the process of designing a distributed system from scratch. Figure 2 is an abstract representation of the traditional system engineering approach in the design of distributed systems. In the early phase of the process, called *System Building Phase*, the goal is to provide a set of properties that the system must satisfy, collectively termed *system assumptions* for simplicity. Their formulation is the aim of the *Requirements Analysis* step (1.), which is arguably the most abstract and also the least formalizable part of the design process. The system designer considers which properties (e.g., safety and liveness specifications) the system shall satisfy and the assumptions and constraints under which they are to be guaranteed. For example, an ATM system will have to respond correctly to clients’ requests, providing money whenever certain conditions are met. Ideally, all these properties and conditions are formulated precisely, completely, and consistently. In reality, however, these specifications are but ideas that shape the system in the very process of making. There are various approaches for requirements engineering, which are typically ad hoc and vary greatly both in their methodology and outcome.

Once the specifications are sufficiently formulated, the actual building of the system begins, usually starting out from higher abstraction levels (*System Design*, 2.) to lower abstraction levels (*Implementation*, 3.). Typically, design approaches are less ad hoc and often supported by tools, in particular, for the automatic translation (compilation) from higher to lower abstraction levels. It is obvious that both the *System Design* and *Implementation* steps heavily rely on the *Requirements Analysis* step: if the requirements turn out to be inadequate, then both the high level design and the implementation will have to change accordingly. Spotting such discrepancies, along with the obvious strive for eliminating design and implementation errors, is in the scope of the *Testing/Verification* step (4.). Here, we enter the *System Analysis Phase* that checks whether the system actually satisfies the desired properties. Ideally, automatic verification techniques such as model checking are used to formally prove that the implemented system satisfies the required properties. In practice, however, manual testing must usually be resorted to.

After the implementation has been tested and the requirements specifications have been verified, which typically involves several iterations of the loop, the system is finally deployed and put into operation (*Deployment*, 5.). What were “ideal” requirements in the system designer’s mind are now implemented in a concrete system, the behavior of which is usually monitored in some way. In the final *System Maintenance* step (6.), errors or other deficiencies detected by means of monitoring, as well as new feature requests, trigger a new iteration of the loop: Changes will be applied where needed, which may, in the worst case, be the *Requirements Analysis* step. In this case, a chain reaction of changes at all lower levels is usually unavoidable.

Of course, this picture is a simplification of what happens in reality, where the process is often much less linear. It should also be obvious that this traditional approach to system engineering potentially facilitates a comprehensive specification of the requirements and the verification of the system correctness, but is very inflexible with respect to changing requirements specifications: accounting for a new feature typically triggers another iteration of the complete system design cycle.

We emphasize that the system assumptions specified by the system designer in the *Requirements Analysis* step must be incorporated in the system in a way that can be satisfied during system operation, and we claim that this process is actualized in the formulation of an a priori* belief set* by the system designer. Interestingly, this happens not only in the early stages of the *System Building Phase*, but continues to play an important role during the whole lifetime of the system:^6^ The life-cycle of a distributed system usually consists of a sequence of iterations of the outer loop in Fig. 2. In fact, each such iteration actually consists of two alternating phases, the *Development Phase* and *Operating Phase*. The Development Phase refers to the first four steps (*Requirements Analysis* to *Testing/Verification*) and is conducted by the system designer; the Operating Phase starts with the fifth step (*Deployment*) and is autonomously performed by the agents. In the rest of this section, we will focus on the Development Phase and on the role of the system designer’s a priori beliefs.

### Elements and structure of the Development Phase

During the Development Phase, the system designer needs to specify the system’s assumptions that will hold in all states of the system, delimiting the set of states that are reachable during execution. This limited set of possibilities must also be provided to agents: in their reasoning, agents will consider only those states of the system that are provided by the system designer. Consequently, agents’ epistemic attitudes are intended to range only over those states provided by the system designer. For this reason, we can call them “agents’ a priori beliefs”, or, to distinguish them from the corresponding notion for the system designer, “*Operational APrB set”*:

**Operational APrB Set (Agents).** *The set of states of the system that are reachable for a given agent. It is provided by the system designer during the development process either via their protocols or by some configuration data.*

Note that the *System Designer APrBs* subsume all the a priori beliefs the to-be-developed system could possibly rely upon, in a format that is accessible to the system designer only, while the agents’ Operational APrB sets can be viewed as a translation (and reduction) of the System Designer APrB into a format that is accessible to the agents. Consequently, the system designer has access to agents’ Operational APrB sets: even if her knowledge of it might not be complete (due to some errors), she can observe the system behavior during monitoring. Thus, agents’ Operational APrB sets constitute part of the a posteriori knowledge of the system designer, as it is used by the system designer to check whether the system functions accordingly. Clearly, though, it is only the Operational APrB sets that will govern the actual runs of the distributed systems after deployment. We will consider the role of the Operational APrB sets in Sect. 4.

The following example demonstrates that the a priori beliefs indeed restrict the states considered possible both by the system designer (System Designer APrBs) and, as a consequence, by the agents (Operational APrB sets).

*Example 1 (Safety property specification)* In a simple traffic light system, a safety property could be informally formulated by the system designer as “the light is never both green and red at the same time.” This a priori belief will then be formalized by the system designer into the agents’ Operational APrB sets. Thus, such a system, while correct, will never reach a state where the variables for green light and red light are both true, meaning that such a state is not present in the set of allowed runs. By enforcing this property, the system considered by the agents is restricted, a priori ruling out certain system states. Note that the situation of having the light both green and red at the same time is nevertheless globally possible: for example, such a violation of the specification can happen in the presence of failures. However, this situation will never be considered possible by the agents in any run because a state violating this safety property is not encompassed by the agents’ Operational APrB sets. This is the reason why fault-tolerant systems amplify the importance of a priori beliefs.

We stress here that a safety property like the one in Example 1 is indeed an element of the System Designer APrBs, which is then transposed into the corresponding formal property in the agents’ Operational APrB sets: it does not depend on any execution, nor is it something that the agents could learn during execution; rather, it restricts the set of possible states the agents consider. One might argue that these a priori propositions are believed by agents within the system, but are known to the external system designer. This, however, does not hold since the system designer can be mistaken in the formulation and/or the implementation of such a property, qualifying it as belief rather than knowledge.

Since the System Designer APrBs determine which variables are relevant and what the agent protocols must do in order to meet the specification, they are compatible with what Tahko refers to as “the a priori principles” (2011 p. 162) grounding the very first a priori* basis* since any correct reasoning of the agents in the system must adhere to it.^7^

**Fig. 3:**
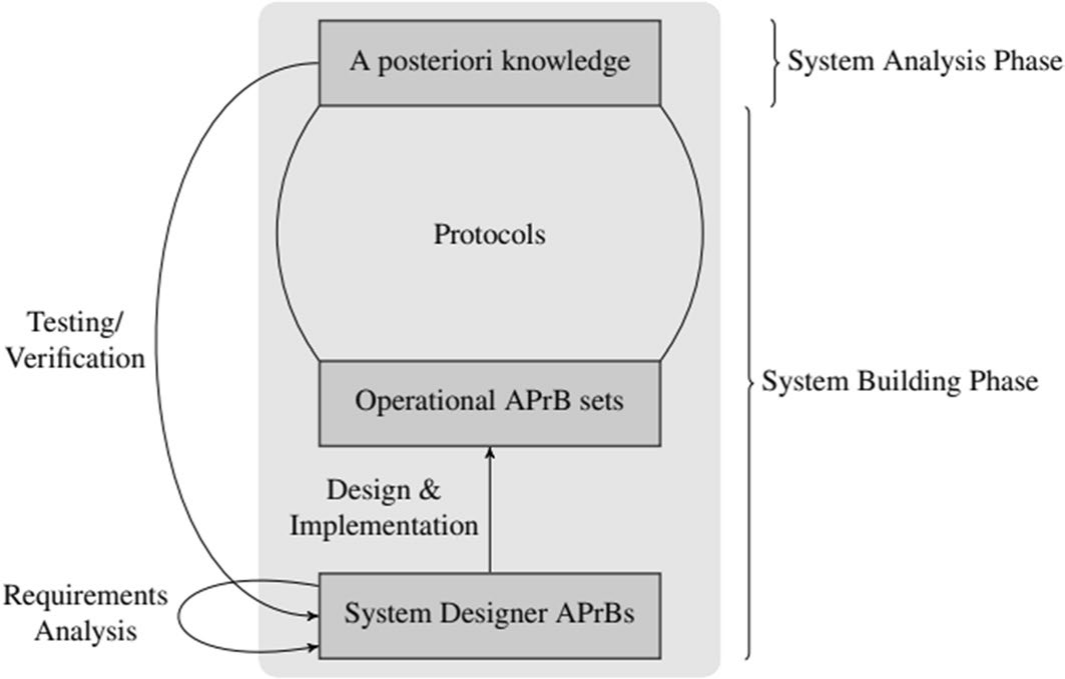
Illustration of the Development Phase of a traditional distributed system. Rectangles depict the various forms of knowledge/belief; arrows represent the steps in the designer process generating them

Figure 3 graphically represents the role of a priori beliefs during the Development Phase: namely, the various forms of belief/knowledge (rectangles) and some of the steps (edges with arrows) involved in its generation. From bottom to top, the figure shows the System Designer APrBs, consisting of the system designer’s a priori beliefs about the system (constraints, safety and liveness specifications, and other properties the system needs to account for) as provided by the *Requirements Analysis* step. The System Designer APrBs are present in the designer’s mind while designing the system and are thus implemented into agents’ protocols, becoming the agents’ Operational APrB sets. As such, the Operational APrB sets are somewhat in between the Development Phase and Operating Phase: they are transposed by the system designer onto the agents in the former, and used by the agents when executing their protocols in the latter. However, whereas the agents do produce (and update) their local a posteriori knowledge in the Operating Phase, as this forms the basis of their actions, they can neither autonomously produce nor update their Operational APrB sets.

In the figure, the arrows on the left indicate the dynamics of the generation of a priori beliefs throughout the Development Phase, which involve a priori* reasoning* by the system designer. Intuitively, a priori reasoning is the dynamic process of formulating and/or modifying a priori beliefs. The arrow going from agents’ a posteriori* knowledge* to the *System Designer APrBs* indicates a priori reasoning occurring during the *Test/Verification* phase: The system designer observes the behavior of the implemented system and records errors and other unwanted behaviors. Depending on the source of the problem, she will apply changes by re-entering one of the steps in the design process. Fixing design and implementation errors may also require revising the Operational APrB sets; fixing specification errors typically additionally requires changes of the System Designer APrBs, necessitating corresponding modifications of the whole system.

On the other hand, the system designer may also intervene at a later stage: namely, during the *System Maintenance*, as depicted by the bottom loop arrow in Fig. 3. This is typically triggered when the system behaves unexpectedly during the Operating Phase, e.g., because there is a severe mismatch between the specification and the monitored behavior or if a new feature has to be implemented. If there is a design or implementation error, the system designer will keep the original System Designer APrBs and will modify only the system design and/or implementation; in some cases, she may also need to adapt the Operational APrB sets. If the system specification is to be changed, however, this inevitably creates the need for an a priori* belief update*—both of the System Designer APrBs and Operational APrB sets —and the corresponding revision of the design and implementation of the protocols. Note that neither the update of the System Designer APrBs nor that of the Operational APrB sets can cause inconsistency problems here: for the former, because we assume a single^8^ designer; for the latter, because all agents start from scratch in the newly built system. It is clear that the exposed a priori belief dynamics involve a bootstrapping relation, in the spirit of Tahko: the system designer, based on newly acquired evidence (that can be gained both during the Development Phase or Operating Phase), discovers that her a priori beliefs cannot guarantee the desired properties of the system; to achieve her goal, she needs to come up with new a priori beliefs and a corresponding a priori belief update.

Evidently, both the Operational APrB sets and System Designer APrBs are purely a priori from the system’s viewpoint^9^ and constitute the basis for the bootstrapping relation executed within the a posteriori framework formed by the system. At the Development Phase, the term “a posteriori* basis*”^10^ subsumes all the information that the system designer has of the system, including the explicit portion of her System Designer APrBs, the Operational APrB sets of agents, and the system itself. In the presence of recalcitrant experience, the a posteriori basis will be revised and will undergo an a posteriori verification step. Consequently, the set of states considered possible by the system designer is fallible and dynamic as it is constantly reformulated based on the feedback given by the system (or a simulation thereof). In addition, the system designer also has some “*background *a priori* beliefs*” that range from propositions relevant to distributed systems to other fields, such as mathematics, philosophy, and so on. For example, some basic logical rules such as the principle of non-contradiction can be considered part of this silent background, as suggested by Tahko (2008, 2011).

### Comparison with Tahko’s account

We conclude this section by comparing the role of a priori beliefs during the Development Phase in the present analysis with the use of a priori knowledge in the philosophical account proposed by Tahko (2008, 2011).

**Fig. 4:**
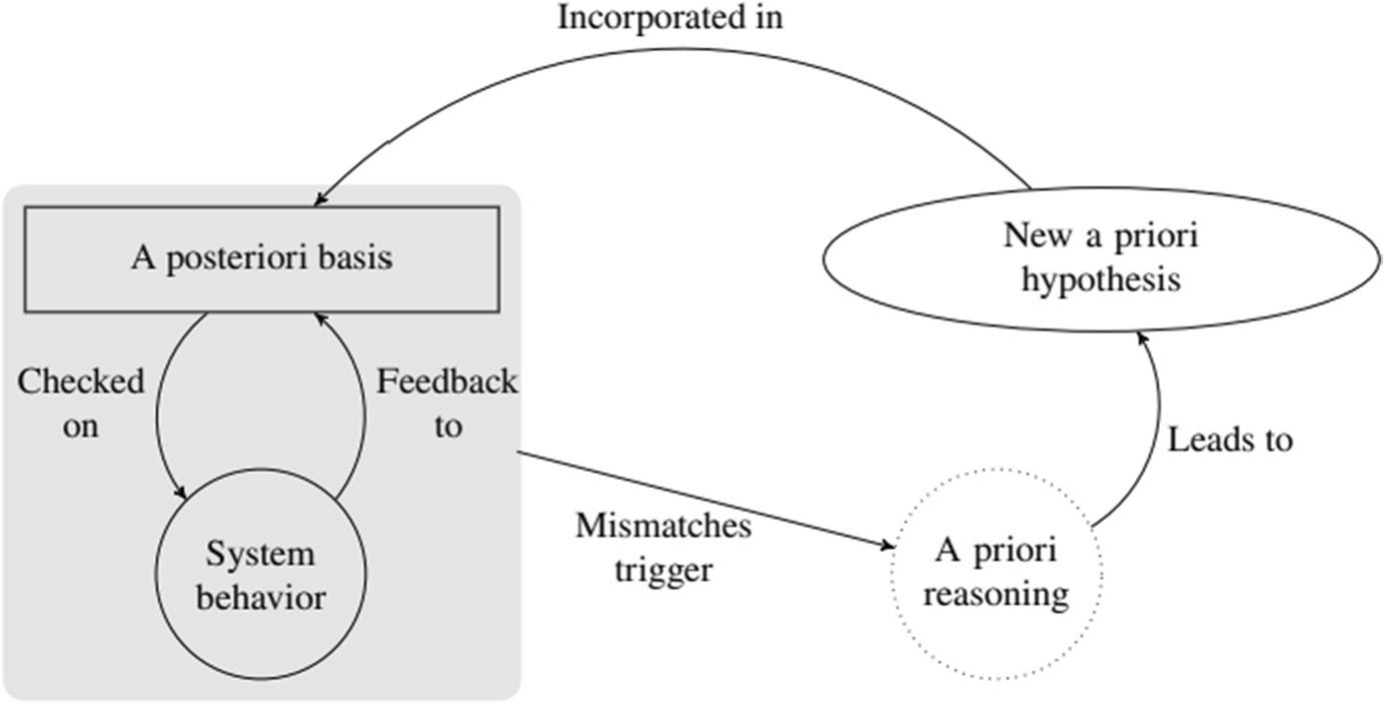
The bootstrapping relation in one cycle of the design procedure of distributed systems

It is apparent that every cycle of the design procedure sketched in the previous subsections indeed involves a bootstrapping relation in the spirit of Tahko (2008, 2011). This relation is captured in Fig. 4.

The *Testing/Verification* (respectively *System Maintenance*) step acts as a checker of the a posteriori base, providing feedback to the system designer regarding whether the requirements and assumptions incorporated in the System Designer APrBs and Operational APrB sets are satisfied. If not, a new Development Phase is initiated where new a priori hypotheses (in the form of new specifications and assumptions) are formulated and a new design and implementation of the system is built.

However, there are some aspects of the present account that are not immediately reconcilable with Tahko’s philosophical analysis. Firstly, the objects and objectives in our context are very different from the classic setting addressed by Tahko, where the primary goal is to check which a priori propositions hold in the only “system” at hand, i.e., in the actual world. In particular, the knowledge of the scientific community about the world is the a posteriori basis against which the actuality of a priori propositions is tested. In man-made distributed systems, however, the situation is rather the opposite: the system designer wants some safety and liveness specifications to hold, under assumptions matching environmental constraints, and thus has to come up with the right system implementation that guarantees those. Hence, the system designer implements (or simulates the implementation of) agents’ protocols according to environmental constraints and has some expectations about her system’s overall behavior. A priori reasoning by the system designer is triggered when her expectations are not matched during (simulated or real) testing. Such mismatches can happen for two main reasons that require separate handling:

(i) The protocols do not agree with the requirement specifications. In this case, it is sufficient to change system parameters and formulate different protocols until the testing and verification stops violating the specifications, without changing any a priori assumptions about the system.
(ii) The target specifications cannot be achieved with the current a priori beliefs, which may have two different reasons: (a) Since the protocols largely depend on the Operational APrB sets, it might be the case that the mismatch is due to an incompatibility between the desired properties and the current Operational APrB sets. To fix this, a revision of the Operational APrB sets is needed: the agents must be equipped with different a priori beliefs in order to achieve the target specification, necessarily influencing the underlying protocols. More drastically, (b) it may be the case that the target specifications cannot be achieved by the current System Designer APrBs: she must come up with different System Designer APrBs, which usually translates into different Operational APrB sets for the agents in the system, which, in turn, will not leave the agents’ protocols and the system parameters untouched. Case (b) is similar to the case of scientific inquiry, with one main difference: While the actual world cannot be changed in scientific inquiry process, in the design of distributed systems not only is it possible to change the agents’ protocols and the system features, but any change to the a priori belief assumptions (being it to the Operational APrB sets or to the System Designer APrBs) will have consequences at the basic system level.^11^

To exemplify the difference between Tahko (2008, 2011) and our view, consider the (very general) a priori proposition “there is no state of the system where *Y* holds.” Taking this proposition in Tahko’s context of scientific inquiry, it could be translated to “in the actual world *Y* does not hold.” The goal of scientific inquiry is to test this proposition, that is to (tentatively) verify the actuality (i.e., truth) of this proposition by means of critical experiments that aim at determining whether *Y* holds. If it turns out that *Y* holds, we cannot just change the world to make *Y* false. The only alternative is to find another a priori proposition that would describe the targeted feature of the actual world more accurately. On the other hand, in the design of a distributed system, in the same situation, the system designer has the option to modify the agents’ protocols (and other system parameters), before changing the a priori beliefs about the system; those will be modified only upon discovery that no protocol, together with the current system parameters and a priori beliefs, can satisfy the target requirements. Crucially, though, the modification of the a posteriori basis at any level has consequences at the underlying levels, resulting in a different system. Consequently, in the present analysis, executing bootstrapping steps does not result in a tree-shaped time evolution of the system as in Fig. 1, in particular, if we ignore the possibility of maintaining multiple versions. After all, we allow the environment and even the desired safety and liveness specifications to just change, which implies that the System Designer APrBs and Operational APrB sets meant to accurately capture those will not just “pile up” in order to match reality better and better, but just change arbitrarily—and so will the implemented system.

## A Priori Beliefs in the Operating Phase

In this section, we describe the role of a priori beliefs during the Operating Phase, where agents execute autonomously while being monitored by the system designer. Thus, the focus is on the Operational APrB sets agents are provided with. We explain how agents use these sets and how this notion relates to the epistemic notion of possible worlds typical of the epistemic analysis of distributed systems.

**Fig. 5:**
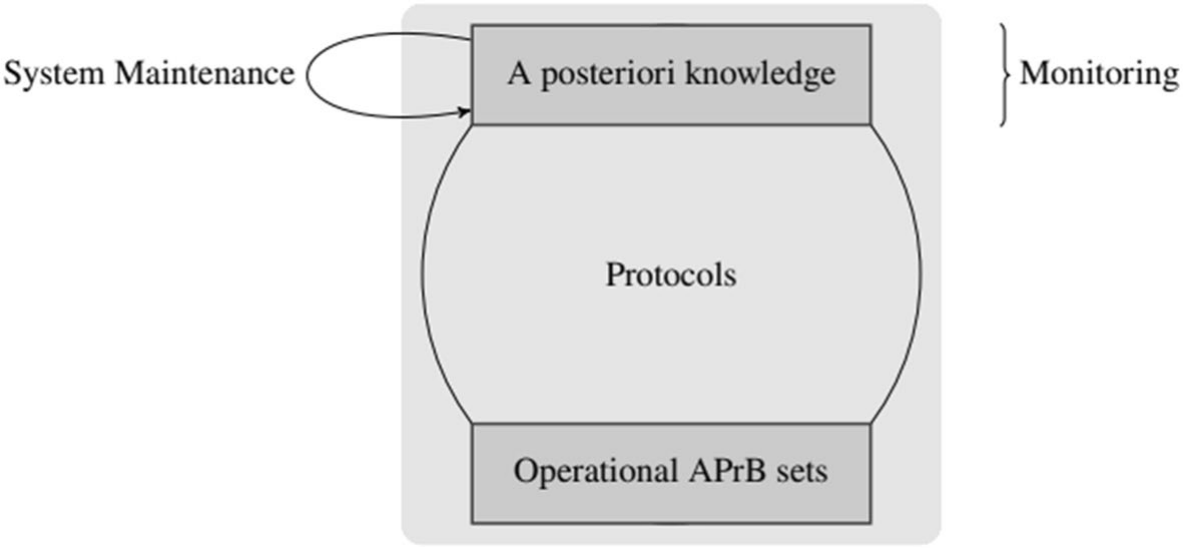
Illustration of the Operating Phase of a traditional distributed system. Rectangles depict the various forms of knowledge/belief; arrows represent the steps in the design process modifying them

Figure 5 illustrates the Operating Phase of a traditional distributed system. Recall that the agents’ Operational APrB sets are provided by the system designer and are made accessible to the agents (via the protocol implementation or via some configuration data) during the previous phase, i.e., the Development Phase. Indeed, the Operational APrB sets remain unchanged throughout the Operating Phase, unless a new cycle of the design process is conducted by the system designer, as described in the previous section and represented by the loop arrow in Fig. 5. Crucially, the a priori reasoning and the update of the Operational APrB sets can be performed solely by the system designer during the (intermediate) Design Phase since agents in traditional distributed systems (unlike SASO systems) lack any introspection on the a priori assumptions governing the executions: when faced with unexpected behavior, they will reach wrong conclusions without hypothesizing about the set of system states that they are provided with. Rather, they just take their respective Operational APrB set as granted and reason epistemically from that basis, even if this leads them to mistaken conclusions. We mentioned already that this does allow even a traditional system to perform some self-adaptation, however, provided these capabilities have been foreseen in the Operational APrB sets.

### Epistemic logic and a priori beliefs

In this section, we elaborate the role of the a priori beliefs in determining the set of possible states typical of the epistemic analysis of distributed systems. In this framework (Fagin et al., 2003), processes are *agents* commissioned to accomplish some task by following a specific protocol. A *protocol* can be viewed as a list of possible actions that an agent may perform in a given situation if certain *preconditions* are satisfied. For ease of exposition, we will focus on *knowledge-based protocols*, i.e., protocols whose preconditions on actions make explicit reference to the local knowledge of the acting agents. Most existing epistemic analyses of distributed systems use the *runs and systems* framework (Fagin et al., 2003; Halpern & Moses, 1990). A system consists of a set of *runs*, each of which models the time-evolution of the global system state, typically formalized as a Kripke model in possible world semantics (Hintikka, 1962). Each possible world stands for a global state of the distributed system and is represented by a *point*, which is a pair of run *r* and time *t*, formally (*r*, *t*). Each agent has access to its *history*, i.e., the collection of *actions* performed and *events* witnessed by the agent, constituting their *local view*, a notion of utmost importance in distributed systems. Agents do not have the global view of the system or of its evolution: they can keep track of only the limited portion of the system they can directly observe, and it is only this portion that is recorded in their history. Only the system designer is assumed to have the global view of the system, even if not necessarily correct and complete. Knowledge is a powerful conceptual abstraction for distributed systems, especially since reasoning about agents’ “states of knowledge” at various points in the execution of a distributed protocol (Halpern, 2003; Halpern & Moses, 1990) has proved to be extremely fruitful both for the designer and the analyzer of a distributed system. Standard epistemic logic relies on a Kripke model *M* that describes the possible global states the agents can be in, where certain atomic propositions (expressing facts like “variable *x* in the state of agent *i* is two”) hold true or false, along with an indistinguishability relation between global states: two points are *indistinguishable* for some agent if and only if it has the same local state at both points. An agent is said to *know* a proposition ϕ (e.g., expressing the value of some variable of the system) at some point (*r*, *t*) if ϕ holds in all points that are indistinguishable from (*r*, *t*) for this agent. This interpretation of knowledge captures the intuition that, being in the global state (*r*, *t*) of model *M*, agent *i* knows ϕ if and only if ϕ holds in every global state (*r′*, *t′*) that is indistinguishable from (*r*, *t*) for *i*. It is easy to see that agents’ knowledge defined in standard epistemic logic terms is a posteriori, as it is gained through observations and reasoning during protocol execution. Related notions such as group knowledge and common knowledge can be defined in the standard way. Formal epistemic modeling and analysis proved to be useful for characterizing, once the system is designed, its evolution over time (Halpern & Moses, 1990), for determining what processes can compute based on their local state in a given model (Ben-Zvi & Moses, 2014; Castañeda et al., 2014; Goren & Moses, 2018; Moses, 2015), and for deriving impossibility results (Moses & Tuttle, 1988). In Kuznets et al. (2019a), a runs and systems framework that models byzantine faulty agents was introduced.

The distinguishing property of the agents’ a priori beliefs is that they do not depend on any particular experience: they are not recorded in the agents’ history, they cannot be inferred from their local states, and they are often not even explicit in their protocols. Still, agents’ a priori beliefs are taken to hold in the runs considered possible by an agent. However, such a priori beliefs may eventually turn out to be wrong, in the sense that there may be runs of the real system that the agent does not consider possible and that violate the intended assumptions, e.g., runs that violate the assumed maximum message delay. Whether the agent is able to detect such a violation or not, its protocols are likely to be inadequate for reaching their goals in such a run. However, so far, no existing epistemic modeling and analysis framework explicitly addresses a priori beliefs and their role in systems like these.

**Fig. 6:**
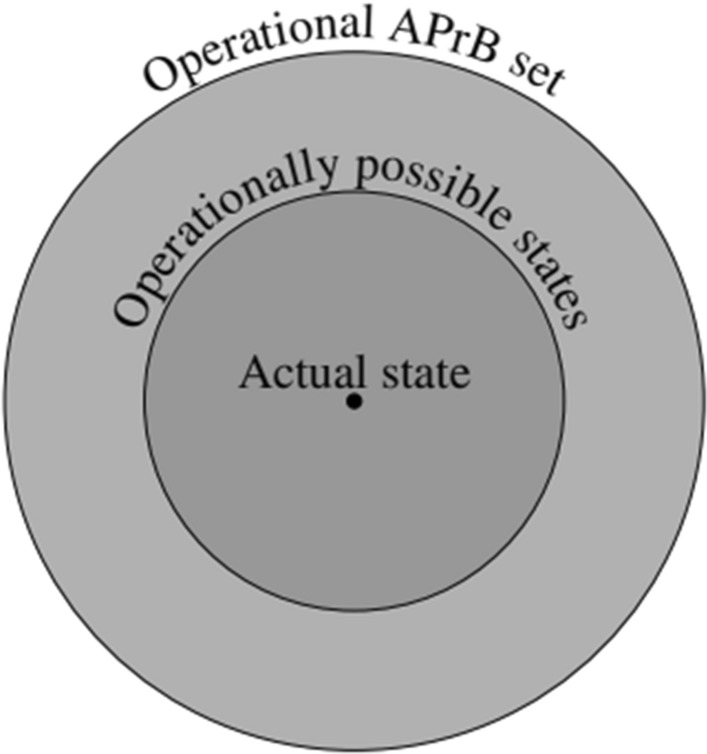
Simple representation of the subset relation between the Operational APrB set and the set of possible worlds involved in a posteriori reasoning. For simplicity, we focus on a single agent only

In order to properly account for a priori beliefs in the epistemic modeling of distributed systems, we advocate that three sets of possible states need to be considered by each agent,^12^ as shown in Fig. 6:

(i) The Operational APrB set, which is provided by the system designer and defines the set of runs and, hence, the set of possible states considered possible by an agent. Since the set of states of a system can be represented by an (epistemic) Kripke model, the (initial) Operational APrB set of an agent corresponds to the initial model that it is considering.
(ii) The *operationally possible* states, that we define as a subset of the agents’ Operational APrB set consisting of the states that are candidates for being the actual state in the current execution. Note that this set is not static: while initially the two sets (i) and (ii) coincide, protocol execution will result in a shrinking of the latter, since the ability of a distributed system to solve some task, like distributed agreement (Lamport et al., 1982), crucially relies on the agents’ abilities to *rule out* certain states as not being the actual state. Fewer states means less uncertainty and thus a gain in knowledge: this process is usually referred to as “epistemic reasoning” in the field of epistemic logic. But since such inferences are made based on local (a posteriori) observations, we will use the term “a posteriori reasoning”. Note that such excluded states, despite not considered valid candidates for being actual, are still a priori possible, since they were defined by the agents’ Operational APrB set. Obviously, the a posteriori reasoning process can only happen within the “space” of possibilities defined by the Operational APrB set; this set of operationally possible states can hence never exceed the set defined by agents’ Operational APrB set.
(iii) The (singleton) set of the actual state, which is (in idealized scenarios, i.e., if the Operational APrB set of that agent is correct) a proper subset of the previous two sets.

**Fig. 7:**
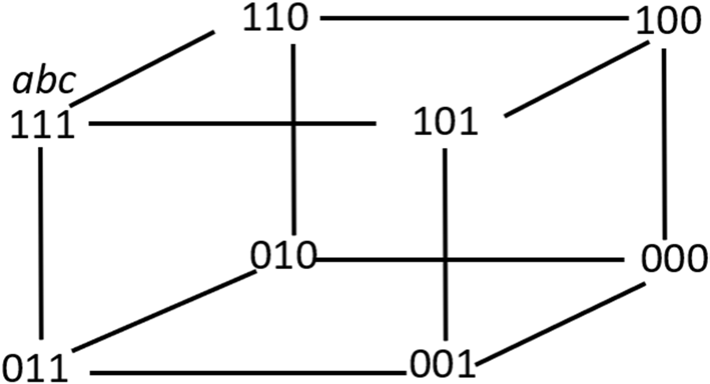
Kripke model representing the set of possible states during a run at time *t* in the simple ATM example. In each state, variables represent parameters in alphabetic order. Lines represent indistinguishability relations. Reflexive relations are omitted

The following examples illustrate this interplay between a posteriori reasoning and the agent’s Operational APrB set:

*Example 2 (Epistemic Modeling of a Simple ATM System)* In the simple ATM example introduced earlier, the set of alternative scenarios could be represented as a Kripke model with eight possible states^13^ that define the set of possibilities considered by the agents: (a) there is a withdrawal request (1) or not (0); (b) all the parameters for accepting the request are met (1) or not (0); and (c) the requested amount is present (1) or not (0), as shown in Fig. 7. In their a posteriori reasoning, agents can build their a posteriori knowledge solely based on this Kripke model and would not consider other scenarios (like providing a different amount of money than the one requested) possible at all.

More specifically, the local a posteriori knowledge accumulated by some agent in the course of the protocol execution represents this agent’s local view of the actual state, which is usually only partial. This a posteriori knowledge determines the protocol actions to be performed next. It depends on the current set of operationally possible states and possibly causes updates to it. Nonetheless, for a state to be considered operationally possible, it must naturally be considered possible first: hence, this subset of states is inherently constrained by the agent’s Operational APrB set. For example, in a conventional ATM system, no agent would consider a state operationally possible where a customer can withdraw a negative amount of money because this is not a possibility foreseen by their respective Operational APrB sets.

*Example 3 (Epistemic Modeling of Muddy Children Protocols)* In the standard formulation of the Muddy Children Puzzle (Fagin et al., 2003), a group of children is playing outside and is called back home by their father. Some of the children have become dirty and have some mud on their forehead. Children can only see whether other children are muddy, but they cannot tell whether they have mud on their own forehead (and it is commonly known to be so). Father then says, “At least one of you has mud on their forehead.” And then, “Those of you that know whether they are muddy please step forward.” If nobody steps forward, father will keep repeating the request. Consider a system where a subset *S* of agents aims at solving the Muddy Children Puzzle. In this setting, each agent in *S* is assigned a boolean variable representing muddiness and (i) no agent knows its own status (being muddy or not) initially, albeit it knows the status of all other agents in *S*, and (ii) the only communication allowed is publicly announcing knowledge/ignorance of one’s status, corresponding to stepping forward/staying put; thus, each agent needs to reach the right conclusion autonomously, based on an environment-regulated round-based event mechanism of public announcements regarding each agent’s knowledge about their own status. The set of initial states encodes all the combinations of muddy/clean agents, forming the Operational APrB sets of the agents in *S*, which determines a corresponding set of runs. However, there are no protocols that would allow agents with these Operational APrB sets to solve the problem. By contrast, it can be solved if the possible state where nobody is muddy is dropped from the set of operationally possible states, which is usually achieved in the puzzle by an announcement originating from a non-agent (father). From any of the remaining initial states, communication can then be used to reliably identify the initial state of the actual run, by gradually discarding (im)possible states from the agents’ sets of operationally possible states. To complicate matters, suppose now that agent *a* in *S* is equipped with a wrong Operational APrB assumption and believes that another agent (say, *b*) is not in *S* while it is. This means that agent *a’*s protocol will ignore agent *b* relative to the Muddy Children task. Hence, albeit *a* may receive messages relevant to the task that are actually sent by *b*, it might reach the wrong conclusion about its own status: this depends on the initial distribution, and thus the correct solution cannot be reached in each possible run. What could happen, however, is that *a* detects that there must be something wrong regarding its Operational APrB set in some run. In any case, what would be needed here for a correct solution would be a way to update the Operational APrB set of agent *a* during a run, a feature that is available only by the intervention of the system designer.

It is also interesting to view the situation discussed in example 3 from the perspective of agent *b* who does know about *a*’s incorrect Operational APrB set: its initial Kripke model in its Operational APrB set is richer than *a’*s model since it includes an additional agent. Besides the fact that the agents no longer have common knowledge of some Operational APrB set, it is important to note the key role of Operational APrB sets in communication: agent *a* is aware of the presence of agent *b* in general, but since it is not considered to be a player in the Muddy Children task, its messages are disregarded or misinterpreted.

More subtle instances of the task involve agents having a “hidden agenda”: they could follow a protocol whose purpose it is to trick other agents into *not* being able to solve the Muddy Children Puzzle in some runs, by exploiting the (simple) epistemic reasoning that underlies the protocols of standard agents. Such considerations led us to the conviction that explicitly incorporating a priori beliefs in an epistemic reasoning framework such as in Kuznets et al. (2019a) is vital for the analysis of byzantine fault-tolerant distributed systems and, crucially, for their ability to tolerate such deviations.

Finally, recall that the system designer might not have complete knowledge of agents’ Operational APrB sets: this is what usually happens in the early phases of design, where the system designer has not yet envisioned all possible states of the system she is designing.

### Comparison with Tahko’s account

The above considerations invite further comparisons w.r.t. the view developed by Tahko (2008, 2011). While there are clear points of connection between the philosophical account and the Development Phase, both the user and the usage of the a posteriori framework in the Operating Phase is very different in the two accounts. In Tahko (2008, 2011), the a posteriori framework provides a basis for testing the actuality of a priori propositions and can be performed by the agent that can also perform a priori reasoning. This view quite nicely fits the situation in the Development Phase, where the system designer indeed uses the a posteriori basis (in the *Testing/Verification* step) to test the appropriateness of the assumptions in the System Designer APrBs. Moreover, it is the system designer who, in case testing uncovers problems, corrects the design or implementation errors or, if needed, performs a priori reasoning in order to figure out new a priori beliefs and applies the corresponding a priori belief update to change inappropriate assumptions in the System Designer APrBs.

In the Operating Phase, however, the situation is very different. The a posteriori knowledge of the agents consisting of their local view of the system is inadequate for testing their respective Operational APrB sets. The best one can hope for here is that an agent detects that there must be something wrong with its Operational APrB set; in a fault-tolerant system, however, it would just (potentially mistakenly) blame itself as having failed instead.^14^ Indeed, agents cannot update their a priori beliefs in the Operating Phase: an update of the Operational APrB set can only be enforced by external means, i.e., by the system designer, in a new cycle of the design loop as described in Sect. 3.1. This is indeed a substantial difference with respect to the account exposed by Tahko (2008, 2011): in the process of scientific inquiry, a priori reasoning may be initiated by the same agent who performs a posteriori reasoning. In traditional man-made distributed systems, the agents do not have such power: they lack the self-reflection and self-awareness tools, as well as a priori reasoning capabilities, to perform Operational APrB updates autonomously.

## Conclusion

We addressed the importance of the role of a priori beliefs in the Development Phase and Operating Phase of distributed systems, emphasizing the different types of a priori states and the different agents concerned. In the Development Phase, the System Designer APrBs, which comprise the system designer’s (still not formalized) view of the system, together with its expected behavior and the assumptions about the environment, are established and used to determine the Operational APrB sets. The latter define the set of possible states of the system (based on the set of relevant variables) and, thus, forms the “arena” in which the a posteriori reasoning of the agents takes place during the Operating Phase.

Throughout our paper, we have also explored the similarities and differences w.r.t. Tahko’s account. We share the view that the a priori is a *modal* notion, albeit concerned with possibility rather than necessity, that it is *fallible*, i.e., that there is no guarantee of correctness of a priori beliefs, and that bootstrapping relations faithfully tie a priori beliefs and the a posteriori reasoning of the agents together. The main differences are the presence of different types of agents: namely, system designer and processes, the ability of the former to affect (“construct”) the actual world, the sometimes different use of the a posteriori reasoning framework, and the fact that the agents do not necessarily have common knowledge of the a priori beliefs.

Regarding future work, our main goals are to incorporate a priori beliefs, a priori reasoning, and model updates explicitly into a byzantine epistemic modeling and analysis framework (Kuznets 2019a). We also plan to extend our considerations to modern adaptive systems, such as the self-adaptive and self-organizing (SASO) systems (Berns & Ghosh, 2009; Tomforde et al., 2014), wherein agents are able to monitor the system behavior and to adapt their view of the system autonomously during runtime. Finally, in the philosophical context, it appears that the restricted context of man-made distributed systems provides a less controversial arena for demonstrating the fruitfulness of Tahko’s original ideas.

## Footnotes

1 Note that in the epistemic analysis of distributed systems, “knowledge” is attributed to agents by the (usually human) system analyzer, based on agents’ limited view of the system.

2 In epistemic logic, factivity is the property of knowledge such that if agent *a* knows *p*, then *p* holds true. Formally, *K*_*a*_* p → p*.

3 Whereas it is typically viewed from the system analyzer’s perspective, a posteriori knowledge also forms the basis of the agents’ actions: Indeed, according to the Knowledge of Preconditions principle (Moses, 2015), if some condition is necessary for performing an action, then knowing that this condition holds is also a necessary condition for performing the action.

4 Gravitational redshift refers to the change in the wavelength of light and other electromagnetic radiation when it travels from a stronger gravitational field to a weaker one (2011 p. 152).

5 We would like to thank an anonymous reviewer for inviting us to explore the connection to Burge’s work.

6 At this point, it seems important to stress that this paper focuses exclusively on “traditional” distributed systems that cannot autonomously change their underlying a priori assumptions during runtime. Whereas they may provide self-adaption features, these need to be explicitly and exhaustively foreseen and implemented by the system designer in the system design phase. By contrast, in proper self-adaptive and self-organizing (SASO) systems (Berns & Ghosh, 2009; Tomforde et al., 2014), the agents are able to monitor the system behavior and adapt their view of the system autonomously during runtime. Interestingly, while the sharp distinction between a priori and a posteriori knowledge characterizing traditional distributed systems exempted us from the need to address Williamson’s concerns (2013), it is not clear whether this is also possible in the case of SASO systems: there is no cleanly separated agency any more, and the obvious interplay between the agents’ experience and their (possibly self-updated) a priori beliefs blurs their clear distinction. However, we leave properly dealing with SASO systems as a topic for future work.

7 Tahko seems to have in mind basic logical principles like the law of non-contradiction, which may provide a foundational a priori basis. We do not need to take a stand on this broader issue here, but the structure of the foundational a priori principles is reflected by the System Designer APrBs.

8 The situation is different if one considers design teams since it often happens that not all designers share a common System Designer APrB set due to inconsistent updates. Modeling this situation is currently outside of our scope.

9 By contrast, from the System Designer’s point of view, the System Designer APrBs typically represent previously acquired (a posteriori) beliefs rather than a priori beliefs.

10 Note that at the initial stage there is typically no implemented prototype system for experimental verification/testing. Nevertheless, some a posteriori verification is already possible by simulations or even model checking, using a coarse initial system model in order to detect specification violations.

11 It is clear that the a priori reasoning step in the bootstrapping relation involves a revision process of the system designer’s beliefs about the system. While the connection with existing belief revision approaches, like AGM (Alchourrón et al., 1985), is immediate, we do not investigate such implementation issues here, as the goal of this paper is solely to develop the philosophical foundations.

12 The picture in multi-agents settings is more complicated, as it requires a distinction between states that are considered possible by that agent and states that are considered possible by that agent to account for other agents’ epistemic attitudes, as well as the representation of the exact relations between agents’ Operational APrB sets.

13 For simplicity, we will consider the initial snapshot at time *t* of a single execution.

14 In fault-tolerant distributed systems, agents can also believe other agents to be faulty. However, in case of a mismatch in its Operational APrB set, an agent has no other option than to consider itself faulty because, in order to consider another agent faulty, there must be a possible state where that agent is faulty. Since there is a mismatch in the Operational APrB set, such a possible state might not exist.
